# *In vivo* efficacy of current stimuli computed by optimal tracking of neuron membrane voltage

**DOI:** 10.1371/journal.pone.0345922

**Published:** 2026-04-29

**Authors:** Alexandra C. Ferguson, Damon A. Miller, John Jellies, Michael Ellinger, Melinda E. Koelling, Cindy L. Linn

**Affiliations:** 1 Formerly with 2; 2 Department of Electrical and Computer Engineering, Western Michigan University, Kalamazoo, Michigan, United States of America; 3 Department of Biological Sciences, Western Michigan University, Kalamazoo, Michigan, United States of America; 4 Department of Mathematics, Western Michigan University, Kalamazoo, Michigan, United States of America; Children#39;s Hospital of Orange County, UNITED STATES OF AMERICA

## Abstract

We describe an application of optimal control theory to *in vivo* intracellular stimulation of pressure-sensitive mechanosensory (P-cell) neurons of the leech *Hirudo verbana*. The control objective seeks optimal stimuli that balance the minimization of stimulation current energy and the error of tracking an action potential evoked by a high-energy rectangular pulse. Tracking a known neuron response mitigates controllability and numerical solution issues and avoids the need to constrain stimulation currents. The reduced second-order neuron conductance model used in optimization was not fit to the target P-cell, but parameters were instead selected based on an assumed saddle-node on invariant circle bifurcation. Optimal stimuli that provided a range of tracking performance and energy minimization were computed prior to experimental work. Remarkably, simulated and biological neurons show the same tracking performance decrease at higher levels of stimulus current energy reduction. Numerical analysis of neuron model responses to optimal current perturbations revealed a phase space separatrix between regions with and without action potential trajectories, demonstrating high sensitivity to optimal current shape in a high-energy reduction case and verifying local optimality. This proof-of-concept study demonstrates that a control strategy based on reproducing action potential shapes can compute reduced-energy current stimulation waveforms that are effective in biological neurons. This effectiveness may extend to other biological neurons since the optimization method was applied to the same reduced-order model for other bifurcations and to a six-dimensional neuron. This method may be useful in research and future clinical applications, particularly as technological advances expand intracellular stimulation to applications previously limited to less effective extracellular methods. The high sensitivity of the neuron response to the optimal current waveform shapes could be useful in drug discovery, neuron disease diagnosis, toxin identification, and provide insights into neuron dynamics.

## Introduction

### Neuron function

Neuron signaling through changes in membrane voltage is the basis of nervous system function. The membrane voltage is maintained and disrupted by the movement of ions, such as potassium and sodium, through transmembrane channels. The equilibrium or ‘resting’ potential is the membrane voltage that balances the flow of ions due to concentration gradients with electric charge repulsion [1]. An action potential is informally defined as an abrupt prototypical change in the neuron membrane voltage from its equilibrium potential. Neurons may ‘fire’ an action potential due to the influence of other interconnected neurons or from sensory input.

Neuron stimulation is a fundamental tool for studying neuron function and for treating conditions including movement disorders, effects of stroke and spinal cord injuries, multiple sclerosis, epilepsy, chronic pain, and hearing loss [2]. There are a large number of neural intracellular and extracellular stimulation techniques. Extracellular stimulation techniques are used in medical devices [3] — intracellular stimulation requires complex experimental procedures [4,5], including insertion of an invasive electrode that eventually compromises the target cell [6,7]. However, intracellular techniques have “revolutionized” understanding of neuron function by enabling direct injection of current through the neuron membrane and direct measurement of membrane voltage [6], both of which are indirectly and inexactly performed in extracellular studies [5]. Miniaturization of electrodes [7,8] and associated electronic circuitry [5] is greatly expanding the reach of intracellular methods, including parallel intracellular membrane voltage recordings from thousands of *in vitro* connected neurons [9] and *in vitro* neuronal control of an *Aplysia* feeding structure [10]. The ultimate goal is development of devices for parallel intracellular electrophysiology for study of large neuronal networks [4] and extension to *in vivo* “clinical neuroscience research” [5].

**Fig 1:**
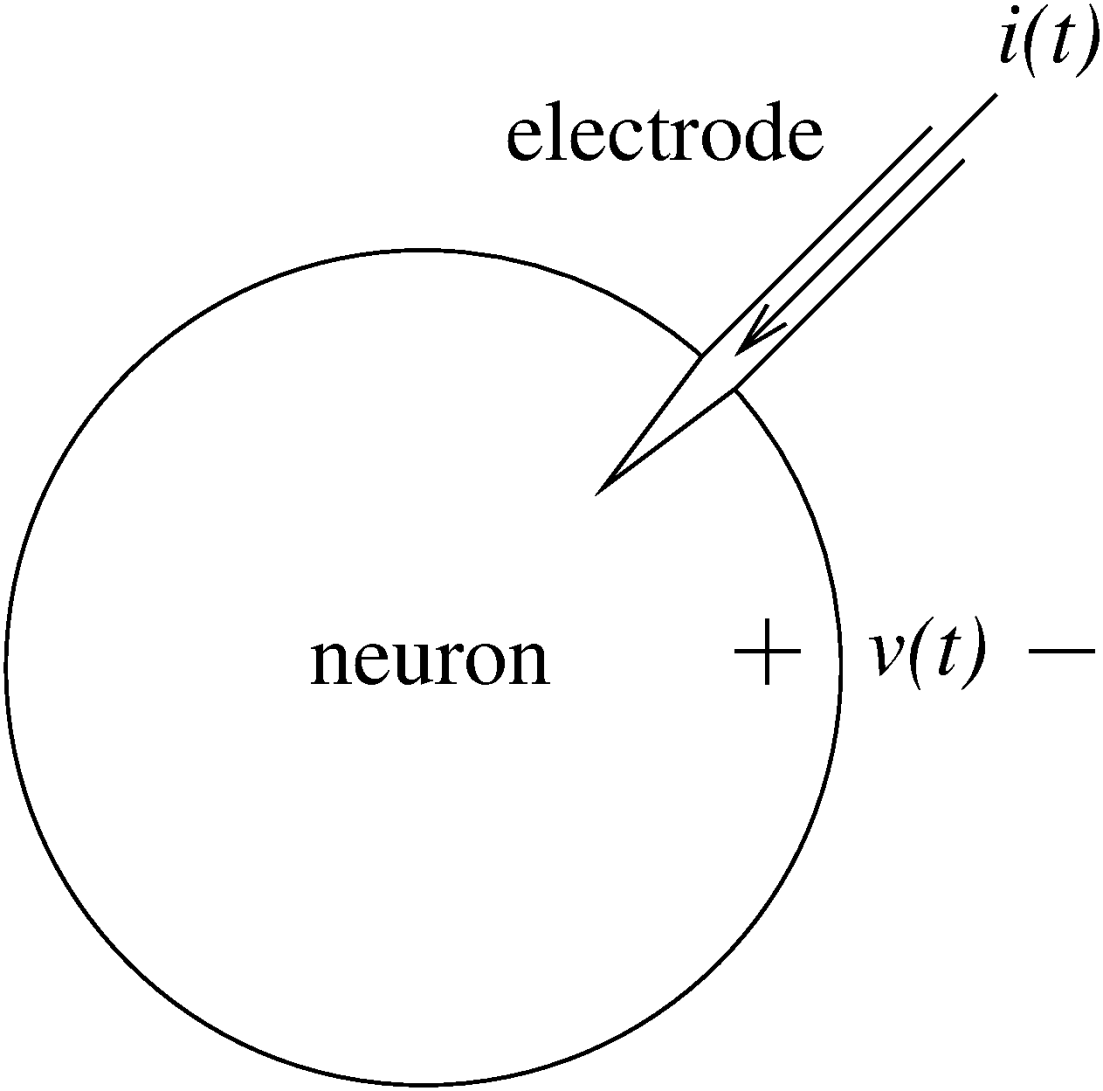
Membrane voltage polarity and stimulation current reference direction. The membrane voltage *v*(*t*) is measured with respect to outside the neuron, in part because the preparation bath is electrically grounded. Stimulation current *i*(*t*) is positive charge flow into the neuron under experimental control. A positive *i*(*t*) tends to increase the membrane voltage, while a negative *i*(*t*) tends to decrease the membrane voltage.

A simplified explanation of neural function is that a neuron produces an action potential if its membrane voltage exceeds a fixed threshold. Exceeding the neuron threshold initiates a cascade of opening and closing voltage-controlled ion channels that quickly change the membrane voltage, generating an action potential. Fig 1 shows the membrane voltage polarity and reference direction for a positive charge flow *i*(*t*) injected into a neuron during intracellular stimulation. We use this commonly-applied convention in this paper. A positive *i*(*t*) tends to increase (‘depolarize’) the membrane voltage, thereby increasing the probability of an action potential. An electrometer connected to the electrode enables direct membrane voltage measurement and direct injection of a specified time-varying current. This current can mimic effects of interconnected neurons on the membrane voltage dynamics, including generating action potentials.

Assuming the neuron firing threshold paradigm, one can plot the stimulus pulse strength needed to produce an action potential versus the stimulus pulse width. A subsequent analysis reveals that there is a pulse width, termed the chronaxie, that minimizes delivered energy for action potential generation, assuming that the stimulation current flows through a constant resistance [11]. A detailed comparison of simulated and experimental intracellular and extracellular chronaxie is provided in [12]. Neural excitability is more formally studied by exploring changes (bifurcations) in neuron dynamics as a function of the applied current in more complex mathematical models. That analysis reveals different mechanisms by which positive or negative stimulation currents can generate an action potential, as observed in biological neurons [13].

*Conductance-based neuron models* as pioneered by Hodgkin and Huxley [14] enable accurate prediction of neuron responses during intracellular stimulation. Conductance-based models consist of a system of differential equations that include nonlinear voltage-dependent ion conductances. Predicting neuron responses to extracellular stimulation is a much more difficult modeling problem [15–18] than intracellular stimulation, owing to lack of direct access to the neuron membrane voltage and indirect stimulus application.

Macroscopic studies of neuronal networks typically analyze action potential timing using temporal and spatial correlations of spikes which are at the core of biological information processing [19]. The shape of the action potential is not important at this level of abstraction. This perspective is reflected in *neuron phase models*, where neurons are treated as nonlinear oscillators to greatly simplify simulation and analysis [20]. The stimulation current changes the phase of the neuron as specified in a phase response curve.

### Optimal control

Achieving a desired output from a system modeled by mathematical equations is a ubiquitous problem in science, engineering, and finance. Optimal control theory provides a well-studied set of methods to not only control a system, but to also conserve actuating inputs. A simple example is minimization of energy used to achieve a certain room temperature. A more complex example is orbiting a satellite in minimal time. Reference [21] provides many such examples. Control problems are usually focused on minimizing a *cost function* that quantifies the ideal system performance. Satellite orbit insertion seeks to obtain and maintain a desired trajectory and is a *tracking problem*. Informally, a system is *observable* if the system states can be measured, and *controllable* if the system can be driven to a desired state. Generally speaking, linear systems have closed-form solutions and can be readily controlled with a variety of classical techniques, usually involving feedback of controlled states [22]. Again generally speaking, nonlinear systems [23,24] do not have closed-form solutions, making control a challenge. Neuron models are typically nonlinear [25].

### Optimal control and neuron stimulation

A brief overview of optimal neuron stimulation provides context for our approach, which has been applied to simulated intracellular stimulation of neuron phase [26] and conductance-based models [27–32]. This approach was subsequently used by another research group for extracellular stimulation [33].

Rectangular current waveforms are commonly used in simulation and experimental neuron stimulation studies. A cathodic rectangular current pulse segment followed by an anodic rectangular pulse segment for charge balancing is typical for clinical applications to mitigate tissue and electrode damage [2]. However, there has been extensive research on exploring non-rectangular waveforms for clinical neural stimulation, with goals of increasing implant battery life, reducing tissue damage, and improving stimulation effectiveness as compared to traditional rectangular pulses [2,34]. A simulation study of several candidate deep brain stimulation waveforms revealed that while rectangular pulses minimized charge injection for intracellular and extracellular scenarios, non-rectangular waveforms minimized stimulation energy in all extracellular cases, and for shorter pulse widths, in intracellular cases as well. That work found that a centered-triangular pulse provided the most energy savings among those compared [2]. Non-rectangular waveforms require less charge to evoke retinal ganglion cell spikes [35].

### Spike timing

Optimal control has been used to find stimuli that evoke spikes at a certain latency or as fast as possible [36], or to desynchronize a neuronal network, with potential implications for clinical deep brain stimulation [37]. Treating action potentials as spikes enables event-based control of spike timing [38,39]. The effectiveness of neuron phase model optimal control is demonstrated in [40], where the resulting charge-balanced minimum energy current stimuli were used to regulate inter-spike-intervals in biological neurons. See [41] for a detailed review of methods to analyze and control coupled neuron oscillators.

### Stimulation energy

Optimal control has also been used to find current stimuli shapes that minimize stimulation energy. This usually requires minimization of a user-selected cost function, e.g., one that measures stimulation current energy. Other criteria include penalizing total charge transferred, failure to generate an action potential, and non-smooth stimuli [42]. An exponentially rising current was shown to be energy-optimal for driving a leaky membrane neuron model of extracellular stimulation to its firing threshold [43]. A Least-Action Principle approach coupled with optimal control provides a globally optimal stimulus to reach threshold in conductance-based models of intracellular stimulation. That method relies on the solution of an ordinary differential equation, rather than a boundary value problem typically required in optimal control approaches and reduces numerical challenges and questions of global versus local optimality [44]. A genetic algorithm found energy and charge-efficient extracellular current waveforms to evoke action potentials in a mammalian myelinated peripheral axon model for individual fibers. Stimulation shapes with and without rectangular pulses for charge balancing were evolved. The efficiency of the resulting Gaussian-shaped curves were verified *in vivo* [45]. A genetic algorithm was also used to find excitatory and charge-balancing current waveforms for cochlear implants. The evolved “non-rectangular biphasic neural stimulation waveform may result in up to 25%” charge and energy savings [46].

### Neuron membrane voltage shape

The neuron membrane voltage shape is often of critical importance; indeed, conductance-based models owe their existence to an inspired analysis of action potential features [14]. Measurements of neuron membrane voltage are often the only experimental data available. There are a myriad number of chemical substances that affect neuron membrane voltage [47]. Analysis of experimentally measured action potential shapes has been used to detect toxins [48] and provides other “valuable information” [8].

Tracking the neuron membrane voltage has been used to control activation and inactivation dynamics in a Hodgkin-Huxley neuron model. By setting the desired membrane voltage below the firing threshold, action potential conduction in a multi-compartment axon model can be suppressed [49]. Membrane voltage tracking can also be used to develop control strategies to produce arbitrary spiking responses [50]. Another optimal control technique can simultaneously estimate neuron states, neuron parameters, and control signals used to improve optimization from limited observations of the neuron membrane voltage corrupted by noise [51].

An ensemble Kalman filter can estimate neuron states and parameters from simulated noisy membrane voltage measurements for control of neuron dynamics [52]. This approach can also be used to control simulated dynamics of an interconnected network of neurons using noisy measurements of the local mean field obtained from photodetectors [53]. Subject to the conditions of observability and identifiability, neuron parameters can be estimated by measurements of neuron membrane voltage [54]. For a survey of these issues, see [55]. A recent approach uses neuron membrane voltage measurements to estimate neuron parameters and ionic currents when the underlying model is known or has errors, with potential application to drug screening [56].

### Stimuli that preserve action potential shape

We have discussed the importance of energy efficient neuron stimulation and other applications of optimal control to neuron dynamics. Our approach to finding reduced-energy current stimuli applies a standard optimal control technique to a reduced second-order conductance-based neuron model to minimize a control objective initially introduced in [27]. The objective simultaneously measures (1) stimulus current energy and (2) ability of the stimulus current to evoke the same time-varying neuron response shape as a high-energy rectangular pulse. The method can also be used to drive the membrane voltage to a specific voltage without regard to tracking intermediate membrane voltages [27].

There are practical benefits of tracking the time-varying membrane voltage produced by the high-energy pulse.

1. Controllability issues are mitigated since the neuron model produced the target action potential shape. In general, nonlinear models cannot be controlled to produce an arbitrary response.
2. Numerical solution of the boundary value problem necessitated by the optimal control method is often difficult and may not converge. The likelihood of successfully computing an optimal stimulation current is increased since the model previously produced the targeted response.
3. The optimized current has approximately the same range as the high-energy pulse since that shape is the initial ‘guess’ for numerical optimization. This eliminates the need to constrain stimulus current extrema.

Yi et al. [33] used this approach for extracellular stimulation by optimally tracking a somatic membrane voltage in a two-compartment neuron mathematical model with the intracellular stimulation current replaced by an applied electric field. The method was also modified to find optimal current stimuli that track a reference phase in an oscillating neuron for two different phase response curves [26].

Our initial work was limited to simulation studies of tracking neuron responses while reducing constant and ramp stimulus current energies [27,28]. Subsequent work included more biologically important pulse stimuli [29–32]. To the best of our knowledge, this is the first proof-of-concept study that confirms that our optimal control objective discovers optimal stimulus currents that are effective in biological neurons, specifically pressure-sensitive mechanosensory P-cell neurons of the leech *Hirudo verbana*. This paper summarizes and builds on our previous experimental work [29–31], particularly [32]. It is critical to note that we are not seeking optimal-energy stimulation currents that simply evoke an action potential; rather, we seek optimal currents that preserve action potential shapes evoked by high-energy pulses. Our study was motivated by the above-mentioned benefits of reducing current stimulus energy, mitigating controllability and observability limitations, and demonstrating that an off-the-shelf optimal control technique can be applied to a biological system.

The Results section describes these key findings.

1. The control objective enables varying the balance between stimulus current energy and tracking error. Increased emphasis on energy reduction decreases tracking performance. This decrease in tracking performance was observed in simulation and experimental results for increasing emphasis on energy reduction of high-energy pulses. The close agreement between simulated and experimental neuron responses to optimal currents is remarkable.
2. A reduced second-order neuron conductance model provides sufficient fidelity to compute constrained-energy optimal currents that are effective *in vivo*. Using a reduced-order model significantly reduces the computational workload of solving the boundary value problem necessitated by the optimal control method and facilitates visualization of results.
3. Results (1) and (2) are even more remarkable considering that neuron model parameters were not fit to the leech P-cell neuron, but were instead selected based on an assumed saddle-node on invariant circle bifurcation (SNIC) mechanism [13] for action potential generation. Recent initial unpublished experimental results show increasing action potential frequency in response to a stimulus ramp current and a lack of subthreshold oscillations, consistent with a SNIC bifurcation.
4. Optimal currents can be computed prior to experimental work based on the assumed SNIC bifurcation.
5. The computed optimal current waveforms exhibit two distinct higher-amplitude regions separated by an area of reduced energy. Intuitively, the initial region initiates an action potential and the final region maintains tracking of the membrane voltage after the action potential peak.
6. Computation of neuron model responses to small perturbations of a computed optimal current reveals a phase plane boundary that separates trajectories that are and are not action potentials. This separatrix suggests that the computed stimulation waveforms meet minimal conditions for action potential production. This same sensitivity predicted in numerical simulations was also experimentally observed.

Experimental data and software required to duplicate all paper results are available at [57] as summarized in *Methods*.

## Results

The control objective [27,28]


$$J[i^{*}(t)]=\underset{\text{tracking error}}{\underbrace{\frac{P}{2}{\left[v^{*}(T)-r(T)\right]}^{2}+\frac{Q}{2}\int_{0}^{T}{\left[v^{*}(t)-r(t)\right]}^{2}dt}}+\underset{\text{stimulus signal energy}}{\underbrace{\frac{R}{2}\int_{0}^{T}[i^{*}(t){]}^{2}dt}}$$ (1)

is the foundation of study simulation results. Optimal control is used to find a stimulus i^*^(*t*) with low-squared area (stimulus signal energy) that evokes a response v^*^(*t*) that tracks a reference membrane voltage *r*(*t*) (tracking error term) over the time interval [0,*T*]. The membrane voltage reference signal *r*(*t*) is *t*he response of a second-order neuron model [13] to a high-energy rectangular pulse. Varying the parameters *P*, *Q*, and *R* in Eq (1) vary the relative impor*t*ance of tracking error and stimulus energy during optimization.

This same approach was used to find optimal stimuli that enable neuron phase models to track a reference phase [26]. The objective functional was used in [33] for optimal extracellular stimulation with an electric field rather than an injected current. Setting *Q* = 0 constrains the tracking term to only the error at time *T*; though not considered here, this is useful for finding currents that drive the neuron to a specific membrane voltage (e.g., the peak of an action potential [27]). The same energy penalty was used in [44] to optimally drive different neuron models to membrane voltage firing thresholds. A similar objective functional that summed membrane voltage tracking and a squared-measure of control effort was used for neuron parameter and state estimation [51].

The functional *J* has no physical interpretation, though *J* can be assigned units [$\mu {\text{A}}^{2}\;\Omega \;\text{ms}/{\text{cm}}^{4}$] in the manner of [43], yielding coefficient units: *P* [$\mu \text{S}\;\text{ms}/{\text{cm}}^{4}$], *Q* [$\mu \text{S}/{\text{cm}}^{4}$], and *R* [Ω]. The last term is proportional to energy for a constant membrane resistance, which is not the case here. The reader is referred to [58,59] for studies of neuron energy flow.

### Optimal current stimuli

**Fig 2:**
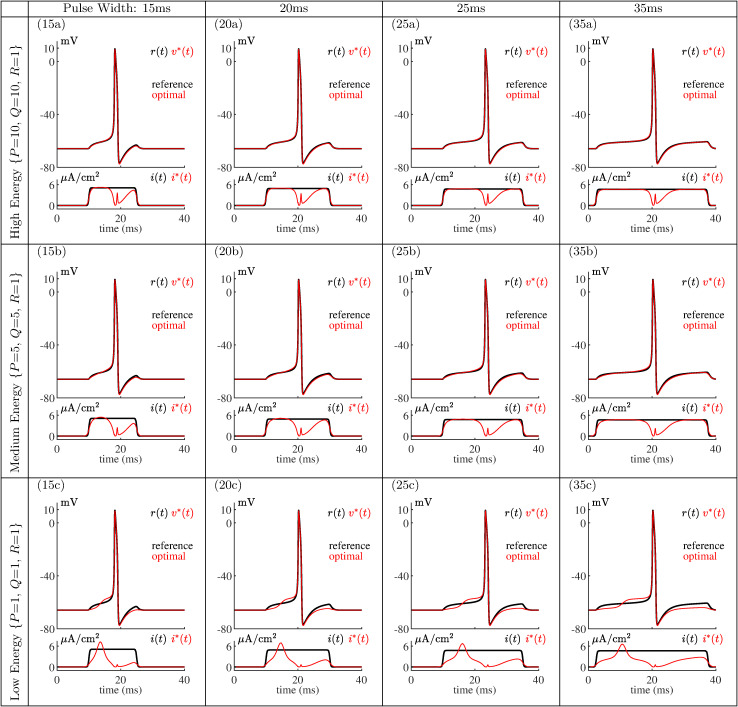
Library of optimal current stimuli. Bottom (each frame): Rectangular pulse stimuli *i*(*t*) (black) and optimal stimuli i^*^(*t*) (red). Top (each frame): Simulated model response *r*(*t*) to rectangular pulse stimuli *i*(*t*) (black) and response v^*^(*t*) to optimal stimuli i^*^(*t*) (red). As *P* and *Q* are reduced, *t*here is a greater emphasis on energy reduction and tracking of the reference membrane voltage degrades.

We generated voltage reference signals *r*(*t*) by selecting rectangular stimulus current pulse amplitudes to evoke action potentials centered within the stimulation pulses to facilitate comparisons of simulation and experimental results. Four biologically relevant pulse widths were arbitrarily selected. We then varied *P*, *Q*, and *R* to place more or less emphasis on energy reduction and computed optimal currents that minimize Eq (1) (*Methods*). Fig 2 shows the resulting library of optimal current stimuli. Each row corresponds to a different emphasis on energy reduction during the optimization process, while each column uses a different pulse width. As optimal stimulus energies are reduced, the tracking error between v^*^(*t*) and *r*(*t*) increases. The spectrum of results range from (top row) very accura*t*e tracking of reference action potentials due to a low emphasis on energy reduction to (bottom row) noticeably less accurate tracking of reference action potentials due to an increased emphasis on energy reduction. The optimal control method discovers current stimulus waveform shapes that produce and track the reference action potentials.

Note that shape and timing of the reference action potential *r*(*t*) is important; indeed, the error between the shape of *r*(*t*) and the actual response v^*^(*t*) in part drives the optimization process. Higher-energy optimal stimuli provide better tracking than lower-energy optimal stimuli. Minimization of functional Eq (1) provides low-energy tracking of the membrane voltage as intended.

**Fig 3:**
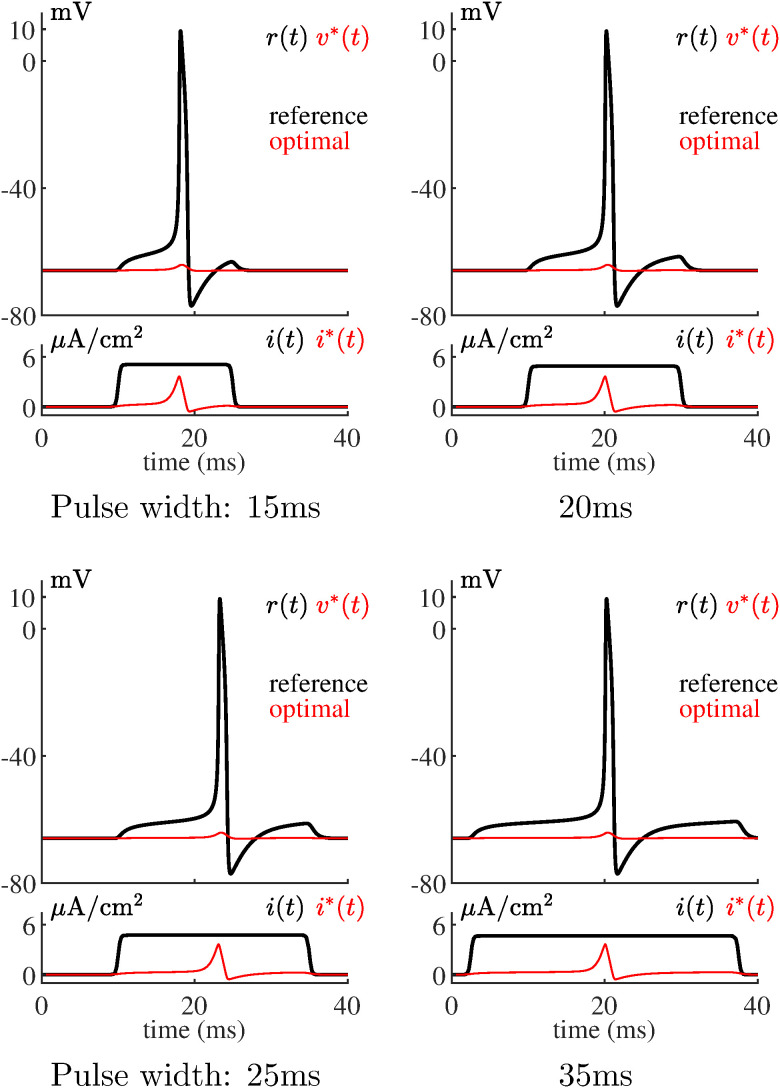
Computed optimal current stimulus and neuron model response for {*P* = 1, *Q*= 1, *R*= 10} and different pulse widths. The model fails to produce an action potential due to the high level of energy reduction.

Setting {*P* = 1, *Q* = 1, *R* = 10} reduces optimal stimulus energy even more than presented in Fig 2. Fig 3 shows that no action potentials are produced by the mathematical model for this level of energy reduction. For these simulations the initial guesses for v^*^(*t*) and n^*^(*t*) were set to resting values over the solution time interval. This verifies that over-emphasis on energy reduction results in a failure to track the target action potential.

### Leech neuron responses to optimal stimuli

We now demonstrate the efficacy of the computed optimal current waveforms in biological neurons. This efficacy is the central study result. The entire current stimulus library of Fig 2 was applied to five different P-cells from midbody CNS ganglia from five different *Hirudo verbana* leeches. Each computed optimal current of Fig 2 was scaled prior to intracellular stimulation by the ratio between the experimentally determined pulse amplitude needed to evoke an action potential approximately centered in the pulse width and the pulse amplitude used in the numerical optimization process. We maintain that five successful experimental trials are sufficient for this proof-of-concept study, particularly given the consistency of results and our rigorous quality benchmarks. Data from a significant number of experiments that did not meet quality benchmarks was discarded (*Methods*).

There are insignificant differences between the optimal currents of Fig 2 and those used in the laboratory. These differences are documented in the software package [57] and result from using a newer version of MATLAB for all paper simulations and/or slightly different solver configurations. The maximum root mean square error difference ranged from 0 to 25nA/cm^2^.

### Responses of one neuron to stimuli

Here we describe responses of one leech P-cell neuron (animal one) to non-optimal rectangular and optimal currents from Fig 2 obtained as described in *Methods*.

**Fig 4:**
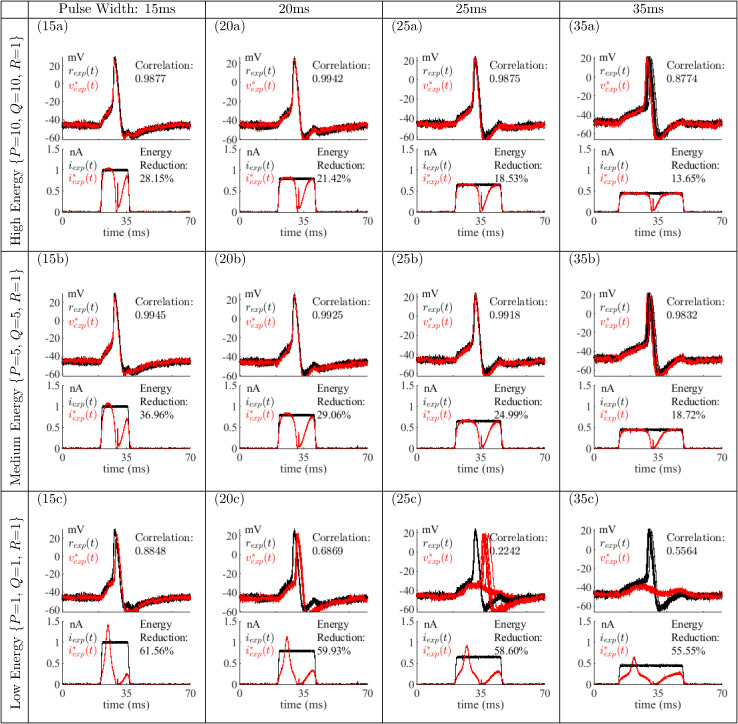
Responses of one leech P-cell to rectangular pulse and optimal current stimuli. Bottom (each frame): rectangular pulse *i*_*exp*_(*t*) (black) and optimal stimuli $i_{exp}^{*}(t)$ (red) as applied to the neuron (ten traces) including percent energy reduction of optimal stimuli. Top (each frame): Responses of P-cell (ten traces) and correlation coefficient of the average *r*_*exp*_(*t*) compared to the average $v_{exp}^{*}(t)$. As the emphasis on energy reduction increases, tracking performance decreases.

Fig 4 shows the single neuron responses to rectangular pulse *i*_*exp*_(*t*) (black) and optimal (red) stimuli $i_{exp}^{*}(t)$. The optimal stimuli are scaled replicas of the waveforms of Fig 2 as indicated by each cell label, e.g., (15a). The correlation between the average responses to rectangular pulse and optimal stimuli was computed using the MATLAB^TM^ routine corrcoef(). Energy reduction is computed by numerically integrating the two stimulus pulses and computing the percent energy reduction of $i_{exp}^{*}(t)$ relative to *i*_*exp*_(*t*). As the pulse width increases, the pulse amplitude required to evoke an action potential centered in the pulse decreases and the action potential timing is more variable. The results for a single neuron are typical across all animals as described in *Responses of five neurons to stimuli*.

Column one of Fig 4 shows results for a 15ms, 1nA rectangular pulse. For the high (15a) and medium-energy (15b) cases there is close qualitative agreement between the action potential, *r*_*exp*_(*t*), evoked by *i*_*exp*_(*t*), and the action potential $v_{exp}^{*}(t)$, evoked by $i_{exp}^{*}(t)$. This is expected given the high emphasis on tracking of the reference action potential during the optimization process. Correlation coefficients close to one confirm the close match between the responses. The energy reduction in the high-energy and medium-energy cases are 28.15% and 36.96%, respectively. A much more substantial energy reduction of 61.56% is achieved in the low-energy case (15c) at the expense of a 1 ms time lag between *r*_*exp*_(*t*) and $v_{exp}^{*}(t)$. This delay is reflected in the lower correlation coefficient of 0.8848.

Column two shows results for a 20ms, 0.8nA rectangular pulse. For cases (20a) and (20b), there is little mismatch between $v_{exp}^{*}(t)$ and *r*_*exp*_(*t*). As expected, the correlation coefficients are close to one and percent energy reductions are 21.42% and 29.06% respectively. For (20c), a 59.93% energy reduction is achieved at the expense of less accurate action potential tracking (correlation coefficient of 0.6869). Note that an action potential is still produced during every sweep in (20c), even though the correlation value between *r*_*exp*_(*t*) and $v_{exp}^{*}(t)$ is relatively low.

Column three shows results for a 25ms, 0.6nA rectangular pulse. In (25a) and (25b) there is little mismatch between $v_{exp}^{*}(t)$ and *r*_*exp*_(*t*), while in (25c) initiation and timing of action potentials is erratic. In (25c), with 58.60% energy reduction, only five of ten optimal stimuli produced action potentials with an average delay of 5 ms. In the other five cases, no action potential is produced. The energy reduction level is so great that five out of ten times, an action potential does not occur, and the other five times there is a delay between *r*_*exp*_(*t*) and $v_{exp}^{*}(t)$, indicating that the selected P, Q, and R values no longer provide good tracking of the reference membrane voltage. This stimulus shape is evidently close to threshold conditions for this pulse width, causing the production of action potentials to be less predictable. This less predictable production of action potentials is common with low-energy stimuli, particularly for longer pulse widths. The correlation coefficient of 0.2242 reflects this behavior.

Column four shows results for a 35ms, 0.45nA rectangular pulse. Variations in the timing of the action potentials are visible even in cases with high-energy content. Yet, a degree of tracking is still qualitatively apparent in the high (35a) and medium-energy (35b) cases. Correlation coefficients of 0.8774 and 0.9832 confirm this observation. In (35c) no action potentials are produced and the correlation coefficient of 0.5564 is much lower. This shows that if energy reduction is over-emphasized an action potential will not occur, as expected.

It is important to note that the energy reduction percentage does not remain constant between different pulse widths for the same values of *P*, *Q*, and *R*. The neuron response is not based solely on energy content, but rather on the pattern in which the energy is delivered. The optimal control method is based on balancing the competing criteria of energy reduction and membrane voltage tracking. For example, in case (20c), the energy reduction is 59.93%, with consistent occurrence of action potentials and a correlation coefficient of 0.6869, while in (25c), the energy reduction is less, at 58.60%, but the neuron produces action potentials less consistently. In (35c) the percent energy reduction is still less at 55.55%, yet no action potentials were produced. It is also important to note that the correlation coefficient can be misleading. In (25c) the correlation coefficient is 0.2242, where the neuron produced an action potential five out of ten times. In (35c), the correlation coefficient is 0.5564 despite the absence of action potentials. Since the correlation coefficient is computed point by point over the entire recorded data, when an action potential is produced late, the correlation of that section of the waveform where it is initiated late is also seen as uncorrelated, making the correlation coefficient further from one.

### Responses of five neurons to stimuli

Here we describe responses of five leech P-cell neurons from five animals to non-optimal (rectangular) and optimal currents from Fig 2 obtained as described in *Methods*, including the animal one neuron responses previously described.

**Fig 5:**
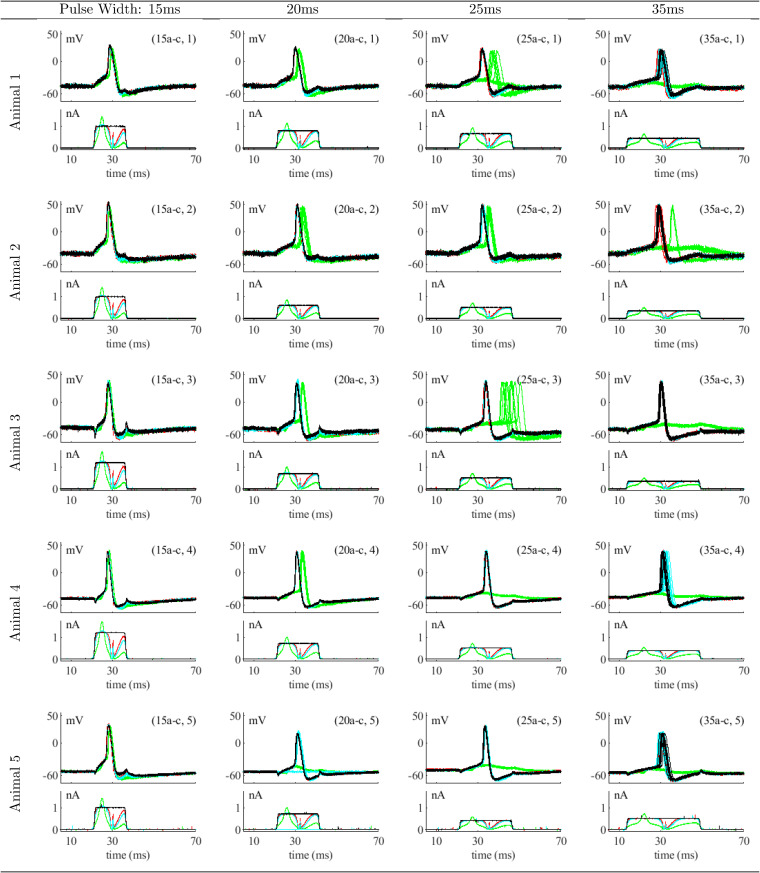
Responses of all neurons to rectangular pulse and optimal current stimuli. Bottom (each frame): rectangular pulse *i*_*exp*_(*t*) (black) and optimal stimuli $i_{exp}^{*}(t)$: high-energy case, red {*P* = 10, *Q* = 10, *R* = 1}, medium-energy case, blue {*P* = 5, *Q* = 5, *R* = 1}, and low-energy case, green {*P* = 1, *Q* = 1, *R* = 1}. Ten stimulus response pairs are shown for each {*P*, *Q*, *R*} case. Top (each frame): responses to rectangular pulse and optimal stimuli in matching colors.

Fig 5 shows the membrane voltage recorded during all sessions. Each cell shows neuron responses to all three energy reduction cases of Fig 2, with response colors coordinated with the corresponding optimal stimuli. For example, case (15a-c,1) corresponds to application of scaled stimuli (15a), (15b), and (15c) from Fig 2 to animal one. Each stimulus is shown in the bottom frame; black is the rectangular pulse stimulus *i*_*exp*_(*t*), red is the highest-energy optimal stimulus ({*P* = 10, *Q* = 10, *R* = 1}), blue is the medium-energy stimulus ({*P* = 5, *Q* = 5, *R* = 1}), and green is the lowest-energy stimulus ({*P* = 1, *Q* = 1, *R* = 1}).

In general the results for neurons from animals two to five mirror those of animal one neuron one. High and medium-energy optimal stimuli provide responses that closely match those provided by rectangular pulses while low-energy stimuli may not, particularly at longer pulse widths.

Results in the 15ms column demonstrate that all levels of energy reduction at this pulse width consistently produce membrane voltage responses which track the reference membrane voltage across all five animals. In contrast, the 25ms column shows variability within a given neuron and across the five animals.

Results for 20ms are shown in column two. Responses to the two highest-energy optimal stimuli show little difference from response *r*_*exp*_(*t*) to the pulse stimulus. With low-energy content (green traces) responses vary. In (20a-c,1), (20a-c,3), and (20a-c,4), evoked action potentials occur at a relatively consistent delay from *r*_*exp*_(*t*). In (20a-c,2) there is greater variability in the delay between traces for this animal. In (20a-c,5), no action potential occurs. This indicates that initiation of action potentials becomes less predictable as energy reduction is increased; the neuron does not always produce an action potential when stimulated.

In column three, with pulse width 25ms, there is much more variability in action potential initiation timing in the low-energy cases, but little difference between the responses to high and medium-energy optimal stimuli and the reference *r*_*exp*_(*t*). In column four, neurons rarely produce action potentials when stimulated with 35ms pulses for the low-energy case — out of fifty traces across five animals, the neuron only produces an action potential two times.

**Fig 6:**
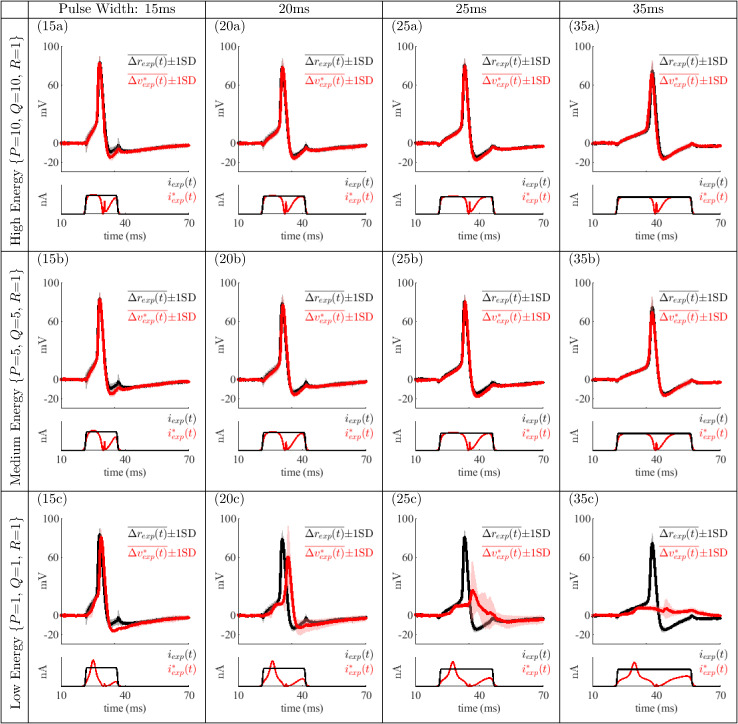
Average and variability of all neuron responses to current stimuli. Each frame shows the average ± one standard deviation (SD) of fifty total responses (across five animals) computed at each sample time after shifting responses to a resting membrane voltage of 0 mV and shifting action potential peaks to the average latency. Bottom (each frame): representative rectangular pulse *i*_*exp*_(*t*) (black) and optimal stimulus $i_{exp}^{*}(t)$ (red) applied to the neuron. Top (each frame): average response to *i*_*exp*_(*t*) (black) showing voltages within one standard deviation of the mean shaded in gray. Average response to $i_{exp}^{*}(t)$ (red) showing voltages within one standard deviation of the mean shaded in pink.

In order to further compare the responses of the five neurons, each frame of Fig 6 shows the variability of neuron responses to pulse and optimal current stimuli across all animals. The average response *r*_*exp*_(*t*) evoked by a rectangular pulse *i*_*exp*_(*t*) is in black with values within one standard deviation of that average response shaded in gray. Likewise, the average membrane voltage response $v_{exp}^{*}(t)$ evoked by scaled optimal stimuli $i_{exp}^{*}(t)$ is shown in red, with voltages within one standard deviation shaded in pink. Since the amplitude of the rectangular pulse *i*_*exp*_(*t*) was varied at the electrophysiology rig to center evoked action potentials within the pulse width for each animal, responses were shifted to a resting membrane voltage of 0 mV. Responses to optimal stimuli for each pulse width case were also shifted to the average latency of action potential peak responses to rectangular pulses across all five animals. These two modifications allow stimuli and responses to be overlaid and compared as shown. Only the shapes of the stimulation currents in Fig 6 are important, since each neuron required a different amplitude of reference stimulus *i*_*exp*_(*t*) to position the peak of the action potential in the middle of the applied stimulus.

The variability of responses to rectangular pulses and to high and medium-energy optimal stimuli is small. For low-energy case (15c), there is a slight delay between the average *r*_*exp*_(*t*) and $v_{exp}^{*}(t)$, but the response variability remains small. For the other pulse widths low-energy cases exhibit poor tracking of the reference action potential.

In (20c), the delay and variability is larger as compared to (15c), and the amplitude of the peak decreased from *r*_*exp*_(*t*) to $v_{exp}^{*}(t)$. The delay and decreased amplitude of the peak of (20c) show that in some trials at this pulse width and level of energy reduction an action potential was not produced. This coincides with a larger variability. In general, these characteristics signify that the initiation of action potentials in the neuron is less predictable.

For (25c) the average response evoked by $i_{exp}^{*}(t)$ does not resemble an action potential or a resting membrane voltage with large deviations. The large variability of the responses shows that the initiation of action potentials is less predictable in this case. At this pulse width and level of energy reduction, in two out of five animals, no action potentials were produced, while in the remaining three animals, production of action potentials was sporadic and always delayed significantly from the reference action potential *r*_*exp*_(*t*). This effect is also evident in Fig 5.

In (35c), the average response indicates that an action potential was rarely produced with a low variability among the responses. There is a slight increase in response variability which indicates some action potentials may have occurred, and indeed, out of fifty pulses, this trial produced two action potentials at an approximate 8 ms delay from *r*_*exp*_(*t*).

A critical observation is that the likelihood of a neuron to produce an action potential in response to optimal stimuli decreases as the pulse width increases, even though the mathematical model, as numerically solved by MATLAB routine bvp5c(), indicated that action potentials should be generated. Numerical solutions provided by MATLAB routine ode45() for cases (25c) and (35c) did not produce an action potential, even though bvp5c() did. Remarkably, this numerical ‘ambivalence’ is reflected in experimental results, and leads to the conclusion that these optimal currents are near a minimal shape for evoking action potentials as discussed next.

### Optimal stimuli separatrix

Minimization of Eq (1) to find optimal currents was accomplished by numerically solving a boundary value problem (*Methods*). In our early work we faced significant numerical challenges in solving the boundary value problem — these difficulties led to our technique of tracking the reference membrane voltage. Given this experience, we decided to verify neuron model responses of Fig 2 using an ordinary differential equation solver. Numerical solution of a boundary value problem (solver bvp5c()) is fundamentally different from numerical solution of a differential equation (solver ode45()). Even so, we would expect that bvp5c() and ode45() would at least predict the same least qualitative response v^*^(*t*) to the same optimal stimulus i^*^(*t*). To our dismay, for cases (25c) and (35c), bvp5c() predicted an action potential and ode45() did not. This subsection describes the investigation of this difference that led us to an important conclusion: small perturbations of the currents computed by bvp5c() can result in very different neuron responses, suggesting the optimality of computed currents.

**Fig 7:**
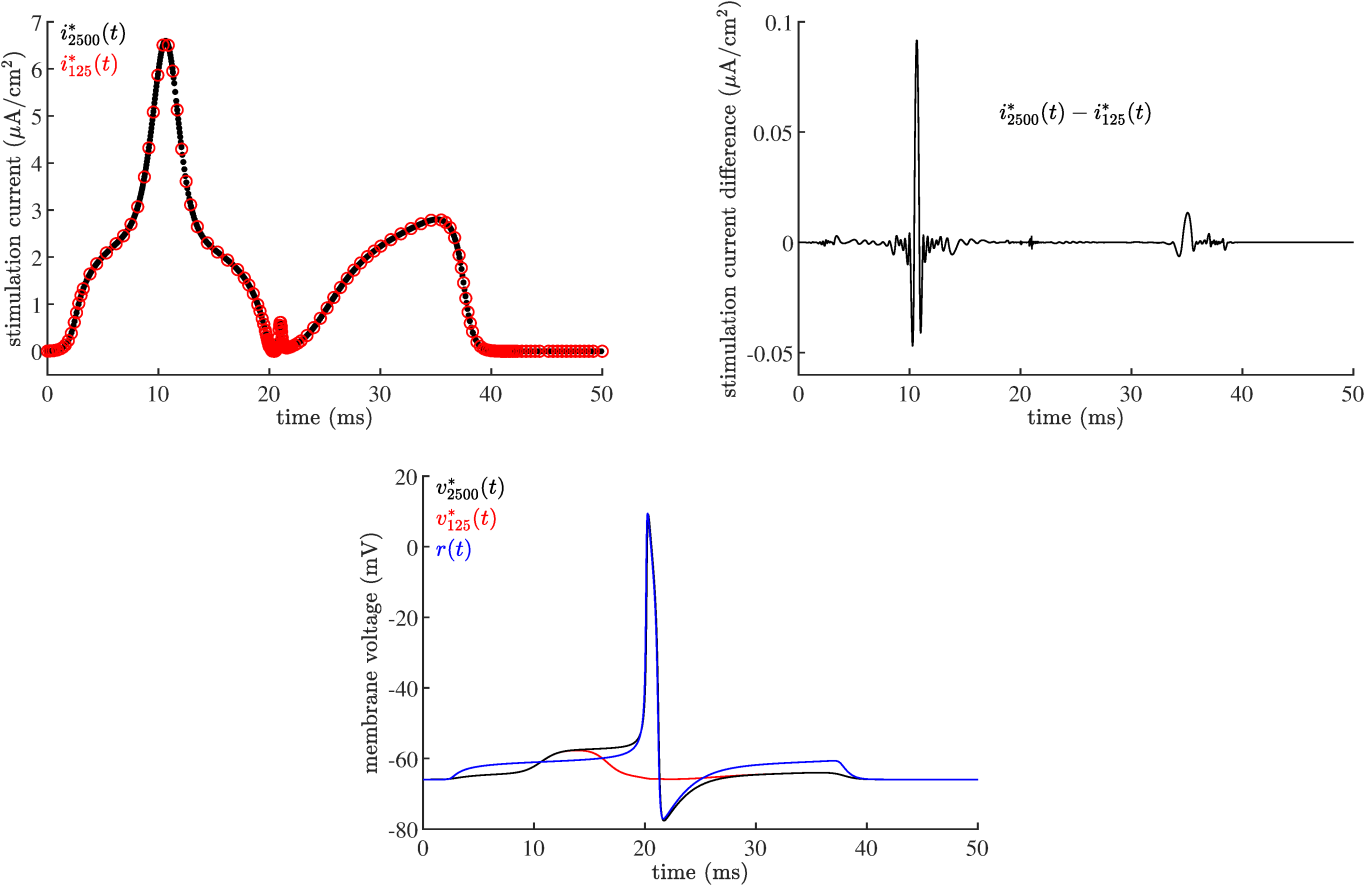
Two optimal current stimuli computed by bvp5c() with different initial mesh sizes. Two optimal stimuli (top, left) $i_{125}^{*}(t)$ (red) and $i_{2500}^{*}(t)$ (black) obtained from bvp5c() by using an initial mesh size of 125 and 2500 points, respectively. The difference between these two solutions $i_{2500}^{*}(t)-i_{125}^{*}(t)$ after interpolation is shown at top right. These two solutions for i^*^(*t*), though quantitatively identical, produce significantly different responses in the neuron model Eq (3) as computed by ode45() (bottom, in like colors). The reference signal *r*(*t*) is also shown for comparison (blue).

Consider the 35ms {*P* = *Q* = *R* = 1} low-energy case (35c) of Fig 2. The reference membrane voltage *r*(*t*) is computed by ode45() using model Eq (3) for a rectangular pulse *i*(*t*). The state *n*(*t*) is also computed during the solution process. The interpolated solutions of *r*(*t*) and *n*(*t*) are subsequently used as initial guesses for v^*^(*t*) and n^*^(*t*) for the boundary value problem solver bvp5c(). Part of configuring the bvp5c() solver is to specify an initial mesh size. Two optimal stimuli $i_{125}^{*}(t)$ and $i_{2500}^{*}(t)$ provided by bvp5c() for an initial mesh size of 125 and 2500 points over the interval [0,100ms] are plotted in Fig 7. The routine bvp5c() predicts an action potential for $i_{125}^{*}(t)$ and $i_{2500}^{*}(t)$; the neuron response computed by ode45() is an action potential for $i_{2500}^{*}(t)$ but not for $i_{125}^{*}(t)$, even though $i_{125}^{*}(t)$ and $i_{2500}^{*}(t)$ are qualitatively identical. The quantitative difference between those two optimal stimuli is very small as shown in Fig 7. Thus one optimal stimuli ($i_{125}^{*}(t)$) results in an action potential using one numerical technique (bvp5c()) but not with another (ode45()), even though a virtually identical optimal stimuli ($i_{2500}^{*}(t)$) results in action potentials for both methods (bvp5c() and ode45()). This indicates that the neural model Eq (3) is extremely sensitive to the shape of the optimal stimulus computed for this case.

**Fig 8:**
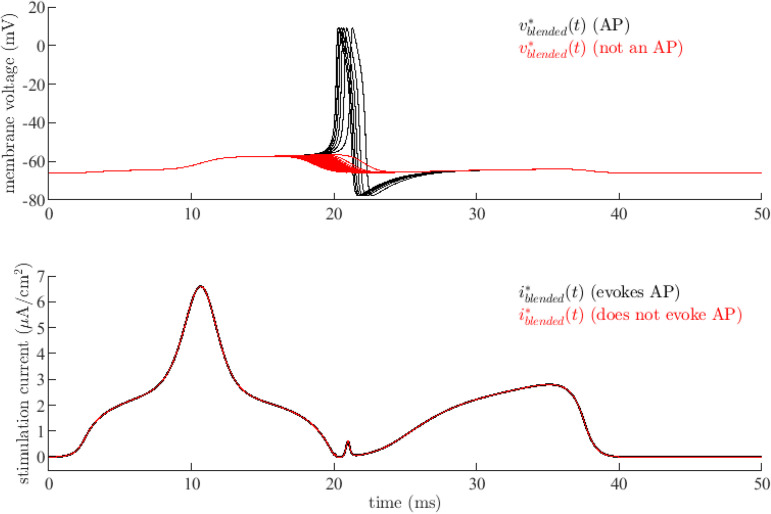
Sensitivity of neuron model response to ‘blended’ optimal current stimuli. Responses v^*^(*t*) of the neuron model Eq (3) (top) to blended currents $i_{blended}^{*}(t)$ Eq (2) (bottom) as computed by ode45(). Small variations in the optimal current create dramatic changes in the membrane voltage response suggesting that this optimal stimulus shape meets minimal conditions for evoking an action potential. Currents that do not produce an action potential and the associated responses are in red.

To explore this sensitivity of Eq (3) to i^*^(*t*), define the ‘blended’ current


$$i_{blended}^{*}(t)=i_{125}^{*}(t)+\epsilon (i_{2500}^{*}(t)-i_{125}^{*}(t))$$ (2)

which is equal to $i_{125}^{*}(t)$ for ϵ=0 and $i_{2500}^{*}(t)$ for ϵ=1. Optimal current interpolations used the MATLAB^TM^ routine interp1() with option ‘pchip’. Fig 8 shows responses $v_{blended}^{*}(t)$ of Eq (3) as computed by ode45() to blended currents $i_{blended}^{*}(t)$ for ϵ∈[0.95,1.0] with $\Delta \epsilon =2.5\times 10^{-4}$. The blended currents produce an increasingly wider period of depolarization, eventually becoming action potentials as ϵ gets closer to 1. Action potential timing is also advanced as ϵ increased, that is, as the blended current approaches $i_{2500}^{*}(t)$.

**Fig 9:**
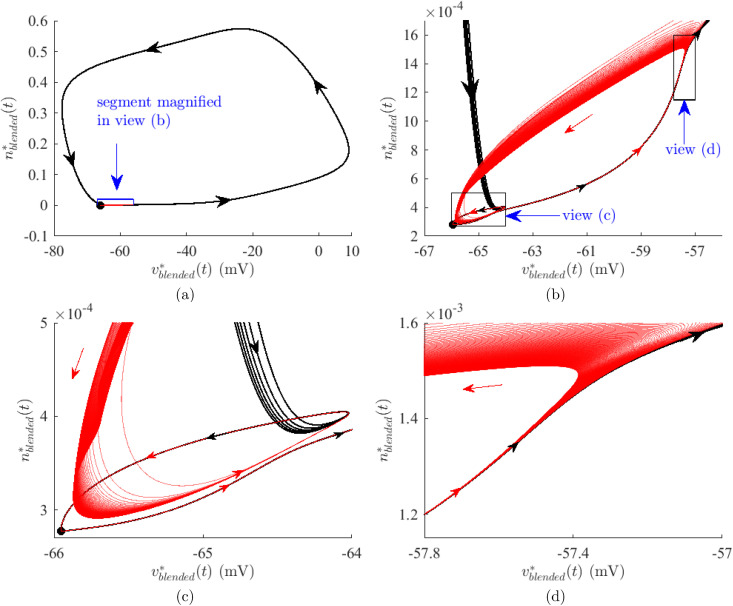
State trajectories of the reduced neuron model Eq (3) evoked by the blended currents of Fig 8. Small changes in the blended current lead to trajectories corresponding to action potentials (black). Those trajectories are separated from trajectories that do not lead to an action potential (red) by a separatrix visible in (d) suggesting that these optimal stimuli satisfy minimal conditions needed to evoke an action potential.

Fig 9 shows state trajectories $\left(v_{blended}^{*}(t),n_{blended}^{*}(t)\right)$ of Eq (3) evoked by the blended currents of Fig 8. The stable equilibrium point found by setting $\dot{v}=\dot{n}=i(t)=0$ in Eq (3) is shown as a black dot in Fig 9(a)-9(c). Trajectories that do not correspond to an action potential are in red. Fig 9(a) is a top level view while Fig 9(b) provides a zoomed view of the boxed area. Note that all trajectories leave and return to the equilibrium point along the same general path. This is further evident in Fig 9(c), a zoomed view of the trajectories near the equilibrium point. Trajectories that correspond to action potentials and trajectories that do not diverge within the boxed area (d) of Fig 9(b). A closer view of that area in Fig 9(d) shows a separatrix that divides the state space into regions leading and not leading to an action potential. This graphically demonstrates the numerical sensitivity of solutions to Eq (3) exhibited in Fig 8. The discovered optimal stimulus $i_{2500}^{*}(t)$ is close to a set of minimal conditions needed to produce an action potential. Further investigation of these minimal conditions is warranted but is beyond the scope of this paper.

This subsection demonstrated that the mathematical model Eq (3) is very sensitive to small perturbations to the low-energy optimal stimuli of Fig 2, at least for cases (25c) and (35c). Looking back at Figs 4 and 5 reveals that this same sensitivity is apparent in real biological neurons for these two cases; that is, in these cases, application of the same optimal stimuli sometimes produces action potentials and sometimes does not. This a testament to both the effectiveness of the optimal control method and the mathematical neuron model.

## Discussion

This proof-of-concept study demonstrates that optimal stimuli found by minimizing a functional Eq (1) [27,28] that balances *tracking error* and *stimulus current energy* are effective at reproducing responses originally evoked by rectangular pulses in pressure-sensitive mechanosensory P-cell neurons of the leech *Hirudo verbana* [29–32]. Optimal stimuli were computed prior to experimental work and a library of stimuli was then scaled for application to biological neurons. For a small sample population of N = 5 P-cell neurons from five different animals, we observed the same spectrum of behaviors in both simulation and experimental work, ranging from accurate tracking of action potentials for high-energy and medium-energy content stimuli, less accurate tracking for lower-energy stimuli, and either intermittent or no action potential production for lower-energy stimuli at longer pulse widths.

### Optimal stimuli shapes

All computed optimal stimuli of Fig 2 exhibit two distinct regions separated by an area of reduced energy, with a higher amplitude in the beginning of the pulse and then a ‘characteristic dip’ in the middle of the pulse. Evidently less energy is required at the end of the stimulation window to lower the error between *r*(*t*) and v^*^(*t*) at or near the end of the action potential. This may reflect channel gating kinetics [60], which could be further explored in the mathematical model by noting the contribution of each ionic current to the membrane voltage.

Stimuli with the highest-energy content are most similar to the reference pulse, but are missing much of the energy in the middle of the pulse. When the energy content decreases to the medium-energy case, stimuli retain the same basic shape, but the corners are more rounded near the edges. Note the excellent tracking performance in the high and medium-energy cases.

Low-energy stimuli have a significant shape change and the peak is higher than the amplitude of the rectangular pulse; this is consistent with known neuron behavior. Both longer lower amplitude pulses and shorter higher amplitude pulses can initiate action potentials. This functionality is discovered and quantified by the optimal control algorithm.

Even though it is unlikely that discovered stimuli i^*^(*t*) are globally optimal given the nonlinearities of Eq (3), the utility of these at least locally optimal stimuli to evoke action potentials is apparent in the results of Figs 4-6, except when stimulus current energy reduction is overemphasized. The mathematical model and biological neurons both exhibit extreme sensitivity to small perturbations in the optimal currents for high-energy reduction cases (25c) and (35c). In simulation, this sensitivity is reflected by the existence of a separatrix in the phase plane for case (35c) as in Fig 9. In the laboratory, this sensitivity is reflected by the failure of some or all neurons in cases (25c) and (35c) to produce action potentials in Figs 4-6. Thus, slight perturbations of a computed optimal current can result in drastically different neuron responses, providing strong evidence for the local optimality of the computed optimal current shapes.

### Neuron model effectiveness

The predictive power of the reduced second-order conductance-based model of Eq (3) from [13] is reflected in the remarkable overall agreement between simulation and experimental results, e.g., between Figs 2 and 4. This agreement is notable not only due to the effectiveness of the low-dimensional model, but also since the model parameters were not tuned in any way, but were instead directly adapted from [13], based on the assumed saddle-node on invariant circle (SNIC) bifurcation type for the target P-cells. Recent initial unpublished results show increasing frequency of action potentials for a current ramp input and lack of subthreshold oscillations, consistent with a SNIC bifurcation [13]. Our experience adds credence to the general recommendation of [13] to focus on the bifurcation type rather than exact parameterization when building a biological neuron model. Also remarkable is the ability of the optimal control method using this neuron model to find optimal stimuli that just meet conditions needed to evoke action potentials, in simulation *prior* to experimental work.

The reduced second-order neuron model Eq (3) can realize four bifurcation types fundamental to neuroscience: saddle node, saddle-node on invariant circle (used in this paper), subcritical Andronov-Hopf, and supercritical Andronov-Hopf by varying its parameters [13]. The optimal control method described in this paper has been successfully applied to each of those four bifurcation types for tracking membrane voltages evoked by current ramps [27] and steps [29]. This suggests that the proposed method has the potential for equal *in vivo* effectiveness for many other neuron types. Also note that the method has been applied to a six-dimensional neuron model [28] and extracellular stimulation [33].

### Open-loop control

From an experimental perspective, this method is open-loop offline control since optimal stimuli were computed prior to lab work; nevertheless, this method discovered optimal stimuli that evoked responses similar to those evoked by high-energy rectangular pulse stimuli in biological neurons. This is further testimony to the model effectiveness as and demonstrates that optimal control of a mathematical neuron model can provide stimuli that can be directly translated to open-loop control in the lab.

### Future work

Our technique of finding optimal currents that track a reference neuron membrane voltage could be useful when reduced energy stimuli are required prior to experimental work or when optimally maintaining the shape of the action potential is of interest. The failure of pre-computed optimal stimuli with modest levels of energy reduction to evoke similar experimental responses could suggest either an inadequate or poorly-fit neuron model. As we have seen, the sensitivity of the neuron model to the optimal current increases as energy reduction is increased. Finding minimal-energy optimal currents that should evoke an action potential across expected neuron model parameter variations might provide a diagnostic tool for identifying abnormal neuron function [8] or environmental toxins [48].

As discussed in the *Introduction*, continuing advances in electrode fabrication and associated electronics are expanding the reach of more effective and energy-efficient intracellular techniques which might eventually include clinical applications [5]. The proposed method reduces current signal energy required to evoke a desired neuron voltage membrane response which could increase implant battery life and reduce net injected charge in future applications.

It should be again noted that the stimulus signal energy term of Eq (1) is *not* proportional to the energy delivered to the neuron except for the case of constant membrane resistance. Ongoing unpublished work is considering adding terms to Eq (1) to penalize the actual stimulator source energy [45] and constrain injected charge [42].

This method might find application to neuronal networks. For example, computing optimal currents that enable tracking the membrane voltage of an interconnected neuron could provide insights into network dynamics and energy efficiency. Another area of interest is extension of this method to extracellular stimulation in a manner similar to [33]. Our technique could be applied to models fit to biological intracellular or extracellular voltage responses evoked by high-energy rectangular current stimuli. Reduced-energy optimal stimuli that preserve the voltage response might be useful in clinical applications, particularly if the stimuli can be computed in near-real time.

## Methods and materials

**Fig 10:**
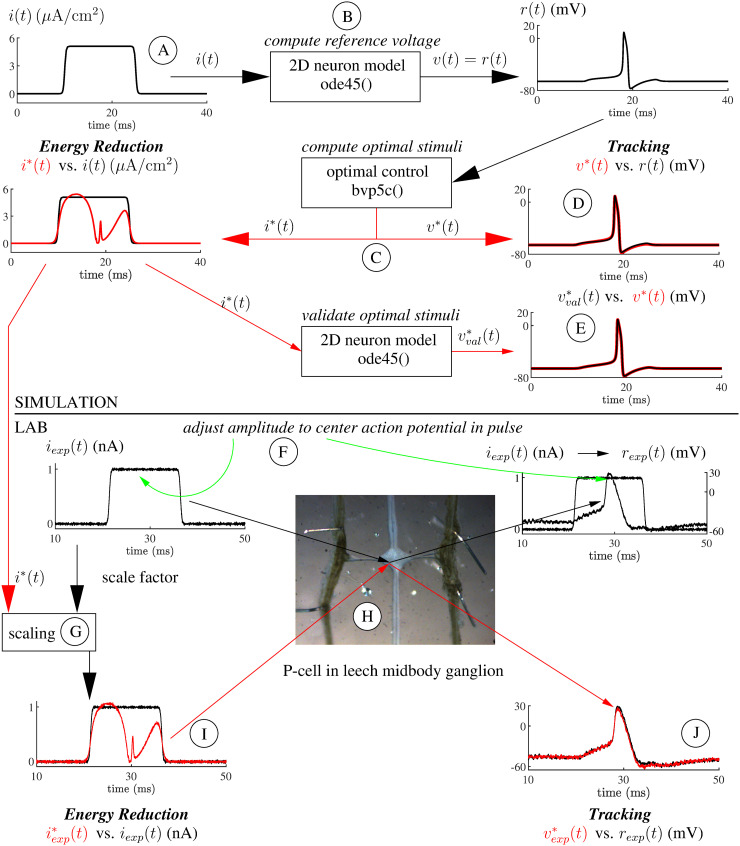
Simulation and laboratory methods summary. Block diagram of the Reduced Energy Input Stimulus Discovery Method [27] as applied to stimulation of pressure-sensitive mechanosensory P-cell neurons in the leech. Circled letters are used in the text to reference parts of the diagram. Photo of leech ganglion courtesy **T.** Groves, Jellies Lab. Waveforms are for illustrative purposes only.

The block diagram of Fig 10 summarizes simulation and laboratory work. Circled letters are used to refer to parts of that figure. Experimental data and software at [57] enable duplication of all paper results.

### Computation of optimal current stimuli

A reduced-order Hodgkin-Huxley type neuron model [13]


$$\begin{array}{l} C\dot{v}=i-g_{\text{Na}}m_{\infty }(v)(v-E_{\text{Na}})-g_{\text{K}}n(v-E_{\text{K}})-g_{\text{L}}(v-E_{\text{L}})\\ \tau \dot{n}=n_{\infty }(v)-n \end{array}$$ (3)

was used in all simulation work, where *v*(*t*) the neuron membrane voltage response to an input current stimulus current *i*(*t*). Parameters *g*_Na_, *g*_K_, and *g*_L_ are sodium, potassium, and leak conductances; *E*_Na_, *E*_K_, and *E*_L_, are Nernst equilibrium voltages; and τ is a time constant. Complexity as compared to the classical Hodgkin-Huxley model has been reduced by elimination of the inactivation variable *h* and assuming instantaneous convergence of activation variable *m*. Steady state values of the gating variables are


$$m_{\infty }(v)=\frac{1}{1+\exp\left(\frac{V_{\text{m}}-v}{k_{\text{m}}}\right)}\text{ and }$$ (4)

$$n_{\infty }(v)=\frac{1}{1+\exp\left(\frac{V_{\text{n}}-v}{k_{\text{n}}}\right)}.$$ (5)

This reduced model is able to realize four bifurcation types fundamental to neurocomputation [13]. Reduced-order models provide the additional benefits of reduced computational times, increased likelihood of boundary value problem solver convergence used in the optimization process, and visualization of system dynamics, as shown with great effectiveness in [13].

Parameters were chosen to provide a saddle-node on invariant circle (SNIC) bifurcation (Table 1 [13]) based on qualitative characteristics of the target pressure-sensitive mechanosensory P-cell leech neurons. Time *t* is in milliseconds (ms) for the the indicated units. Initial conditions *v*(0) and *n*(0) were set to the stable equilibrium point $\approx (-65.95\;\text{mV},2.77\times 10^{-4}\;\mu \text{A}/{\text{cm}}^{2})$.

**Table 1:** Neuron model parameters [13].

*C*	τ	*g*_L_	*E*_L_	*g*_Na_	*E*_Na_	*g*_K_	*E*_K_	*V*_m_	*k*_m_	*V*_n_	*k*_n_
($\mu \text{F}/{\text{cm}}^{2}$)	(ms)	(mS/cm^2^)	(mV)	(mS/cm^2^)	(mV)	(mS/cm^2^)	(mV)	(mV)	(mV)	(mV)	(mV)
1	1	8	−80	20	60	10	−90	−20	15	−25	5

Pulse stimuli used in simulation and experimental work are smoothed pulses of the form


$$i(t)=(a/2)*(\tanh(m*(t-t_{d}))-(\tanh(m*(t-t_{d}-p))))$$ (6)

where *a* is the amplitude of the current stimulation pulse, *t*_*d*_ is the pulse delay, *p* is the pulse width, and *m* = 3.5 ms^-1^ determines the steepness of the transition from 0 to *a*. Smooth transitions guarantee unique solutions of differential equations and reduce electrophysiology stimulation artifacts.

Varying the parameters P, Q, and R in Eq (1) alter the balance between tracking error and stimulus energy minimization. Note that Eq (1) is in the form prescribed by the “Continuous Nonlinear Optimal Controller with Function of Final State Fixed” method of [21]. Signal *r(t)* is the neuron response to a rectangular pulse stimulus *i(t)* Ⓐ found using the MATLAB™ routine ode45() Ⓑ. The pulse amplitude was selected to approximately center the action potential in the pulse width.

Optimal stimuli i^*^(*t*) were found by numerical solution of an associated boundary value problem using the MATLAB^TM^ routine bvp5c() Ⓒ. Previous work used bvp4c() [27–29]. Refer to [27] for the boundary value problem equations and important numerical considerations. Routine bvp5c() not only provides i^*^(*t*), but also an estimate of the neuron model response v^*^(*t*) to i^*^(*t*). For perfect tracking v^*^(*t*) matches *r*(*t*) Ⓓ. As a check, ode45() is used to compute the response v^*^(*t*) to the current i^*^(*t*) — that result should match the output of bvp5c() v^*^(*t*) Ⓔ. This validation was in general successful, except for some notable exceptions. Those exceptions led to impor*t*ant insights into the optimality of the computed optimal currents as discussed in the paper.

### Electrophysiology

#### Preparation.

Experiments to validate the biological efficacy of pre-computed optimal currents i^*^(*t*) were performed using P-cells within midbody CNS ganglia of the leech *Hirudo verbana*. Each ganglion has a typical arrangement of neurons enabling repeatable targeting of specific neurons from one animal to the next [61]. Midbody ganglia were dissected from an adult leech and pinned in a separate dish filled with Ringer’s solution [61] and electrically grounded.

#### Electrophysiology rig.

As in [29–32], a World Precision Instruments Duo 773 Electrometer was controlled by a custom LabVIEW interface to apply stimulation currents and measure membrane voltages in balanced bridge mode. A micropipette was pulled using a Sutter Flaming-Brown micropipette puller and filled with 1M potassium acetate. The electrode was used to impale a P-cell in the selected ganglion using a micromanipulator. Electrode resistance was not recorded but is typically 40-50MΩ. Signals were acquired at a sample rate of approximately 44.1kHz using a NationalInstruments USB-6211. All time values in subsequent computations and plots assume that sampling rate. Since the USB-6211 uses a single multiplexed digital-to-analog converter, signals were not simultaneously sampled. The maximum delay between measurements considered to be simultaneous is about 17μs, an insignificant delay in this context. The LabVIEW interface provides a trigger signal which is recorded in the experimental data, specifies the stimulation current, and measures the DUO 773 current monitor and bridge output (the neuron membrane voltage). The USB-6211 and corresponding circuitry is housed in an aluminum enclosure to reduce electrical noise.

### Stimulation protocol

The neuron resting membrane voltage was recorded for a zero stimulation current before and after each experiment. A small stimulation current between approximately −11 pA and 16 pA was observed when a zero stimulation current was specified, indicating a slight DC experimental offset. The resting membrane voltage was verified to be unchanged after application of all signals.

As in simulation, the amplitude of a rectangular pulse *i*_*exp*_(*t*) was adjusted to evoke an action potential *r*_*exp*_(*t*) approximately centered in the stimulation pulse width Ⓕ. Pulse widths were 15ms, 20ms, 25ms, and 35ms. The rectangular pulse *i*_*exp*_(*t*) was generated within LabVIEW using Eq (6) and applied ten times. Both stimulus and response were recorded.

Next, each optimal stimulus i^*^(*t*) was scaled by the ratio of the amplitude of the pulse *i*_*exp*_(*t*) found in the previous step to the amplitude of the pulse *i*(*t*) used to generate i^*^(*t*) (Ⓖ). The resulting optimal stimuli $i_{exp}^{*}(t)$ were successively applied to the target neuron and the response $v_{exp}^{*}(t)$ recorded ten times Ⓗ (nine instead of ten recordings were made for three of eighty experimental sessions. Thus the total number of recordings is 797 instead of 800. For simplicity this detail was ignored in *Results*). The culmination of the experiment is to note the tracking performance (compare $v_{exp}^{*}(t)$ and *r*_*exp*_(*t*) Ⓙ) provided by the level of stimulus energy reduction (compare $i_{exp}^{*}(t)$ and *i*_*exp*_(*t*) Ⓘ). Each experimental stimulation session required about thirty minutes once the leech ganglia were removed and prepared for the procedure.

**Data Quality Benchmarks** After applying the optimal stimuli for a single pulse width, the rectangular pulse *i*_*exp*_(*t*) was applied again to compare the neuron response before and after the optimal stimuli have been applied. If the response to the rectangular pulse from ‘before’ and ‘after’ differed qualitatively, the data was discarded, assuming that either the electrode or cell impalement conditions changed during the experiment. Data was collected for zero current stimulation after all signals had been applied. If the resting membrane voltage shifted by more than 10% from ‘before’ to ‘after’, the neuron was assumed to have changed significantly during the experiment and the data was discarded. Thus, there were two benchmarks of quality to assure experimental data integrity. Data from a significant number of experiments that did not meet these quality benchmarks were discarded.
